# Aβ42 as a Biomarker of Alzheimer’s Disease: Is Saliva a Viable Alternative to Cerebrospinal Fluid?

**DOI:** 10.3390/brainsci12121729

**Published:** 2022-12-17

**Authors:** Silvia Boschi, Fausto Roveta, Alberto Grassini, Andrea Marcinnò, Aurora Cermelli, Fabio Ferrandes, Innocenzo Rainero, Elisa Rubino

**Affiliations:** Department of Neuroscience “Rita Levi Montalcini”, University of Torino, 10126 Torino, Italy

**Keywords:** saliva, CSF, beta amyloid 42, Alzheimer’s disease, biomarkers

## Abstract

The identification of reliable biomarkers in biological fluids is paramount to optimizing the diagnosis of Alzheimer’s disease (AD). Measurement of Aβ42, t-tau, and p-tau in cerebrospinal fluid (CSF) is the most accepted method to support the diagnosis of AD. However, lumbar puncture represents an invasive investigation, whereas saliva is one of the most accessible body fluids. The aim of our study was to investigate salivary concentrations in AD and evaluate the correlation between salivary and CSF Aβ42 concentrations in AD patients, patients with non-AD dementias, and controls. We recruited 100 subjects: 18 AD patients, 64 patients with non-AD dementias, and 18 controls. The mean saliva Aβ42 concentrations in AD patients were higher than in controls (*p* < 0.001), and to patients with non-AD dementias (*p* = 0.001). A significant negative correlation between salivary and CSF Aβ42 concentrations was found in the overall group (r = −0.562, *p* < 0.001) and in non-AD patients (r = −0.443, *p* < 0.001). Salivary Aβ42 concentrations positively correlated with CSF t-tau (r = 0.321, *p* = 0.001) and p-tau (r = 0.297, *p* = 0.001). Our study showed that in AD patients’ saliva, Aβ42 concentrations are specifically increased, and we found an interesting negative correlation between CSF and salivary Aβ42 concentrations that warrants further investigation.

## 1. Introduction

Alzheimer’s disease (AD), the leading cause of dementia, is a neurological condition that leads to loss of independence, diminished quality of life, premature mortality, caregiver burden, and high levels of healthcare costs. AD is characterized by the progressive deposition within the brain of several misfolded proteins [1]. Senile plaques and neurofibrillary tangles are the main pathological hallmark of the disease and are related to the progressive deposition of amyloid-β and phosphorylated tau proteins [2].

The identification of an accessible and non-invasive test that can demonstrate in vivo the neuropathological hallmarks of Alzheimer’s disease is a major scientific goal in this research field. Cerebrospinal fluid (CSF) tests for amyloid-β (Aβ42 or Aβ42/40 ratio), phosphorylated tau (p-tau), and total tau (t-tau) have been incorporated into the biological definition of Alzheimer’s disease proposed by the National Institute of Aging and the Alzheimer’s Association (NIA-AA) [3]. However, the invasiveness of the lumbar puncture is one of the main limitations to its widespread use in clinical practice. This is driving research efforts towards the validation of alternative tests on more easily accessible body fluids. Although the main scientific interest is directed towards identifying AD blood biomarkers, a growing body of evidence suggests saliva as a rich source of potential biomarkers that could offer some advantages [4].

Most studies on salivary biomarkers in AD have focused on the assessment of Aβ42 [5]. The first study quantified salivary and plasma concentrations of Aβ40 and Aβ42 in AD patients and controls and showed significantly elevated Aβ42 levels in the saliva samples of AD patients [6]. Further studies confirmed an increase in salivary Aβ42 concentrations in AD patients when compared with controls [7] and that this increase appeared to correlate with disease severity [8]. In one study, on the other hand, salivary Aβ42 was found to be slightly reduced in AD compared with controls [9], whereas two studies failed to quantify Aβ42 in saliva [10].

**Figure 1:**
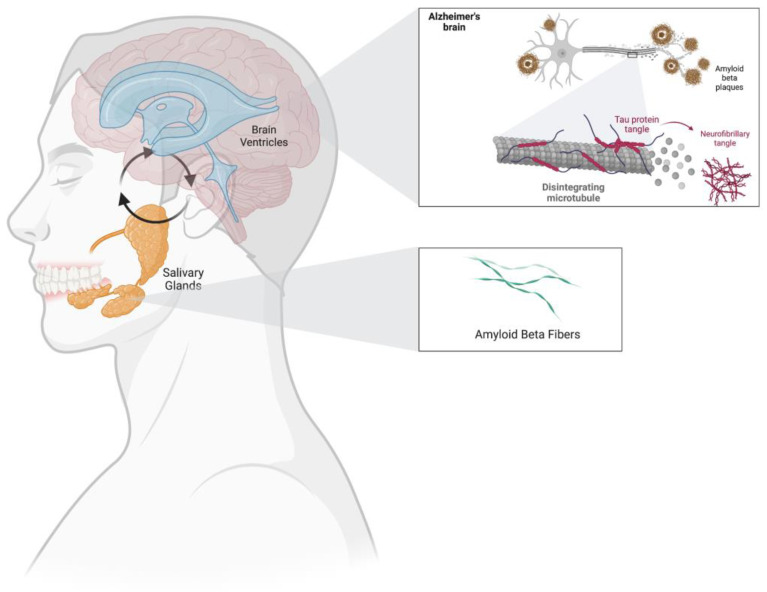
The hypothetical interaction linking the pathological processes of Alzheimer’s disease at the brain level and pathological amyloid-β fragments at the salivary level. This figure was made with Biorender.com.

In the majority of these studies, no significant differences were found when comparing salivary Aβ40 concentrations between cases and controls. Some studies also investigated salivary t-tau and p-tau as AD biomarkers [11], showing conflicting results for salivary p-tau and negative results for salivary t-tau [12]. To date, there are no studies involving AD patients and controls that have investigated Aβ42 concentrations in both CSF and saliva. How a fluid such as saliva would reflect changes in the Central Nervous System (CNS) processes remains to be elucidated (Figure 1). 

The purpose of this study was to investigate salivary concentrations in AD patients and evaluate the possible relationships between CSF and salivary concentrations of Aβ42 in a population of subjects consisting of patients with AD, patients with non-AD dementia, and a control group.

## 2. Materials and Methods

### 2.1. Subjects

We included in this study 100 consecutive subjects (45 males and 55 females, mean age: 69.23 ± 8.35 SD years) referred to the Aging Brain and Memory Clinic at the Department of Neuroscience of the University Hospital “Città della Salute e della Scienza” of Turin, Italy.

Patients underwent lumbar puncture on suspicion of early-stage dementia and were then classified according to the NIA-AA 2018 criteria [3]. Following the “A-T-N” classification scheme through the analysis of the core Alzheimer’s disease CSF biomarker profile (Aβ42, p-tau, and t-tau), the acquisition, in selected cases, of an amyloid PET scan with flutemetamol. Patients were subsequently categorized as being part of the Alzheimer’s continuum (hereafter referred to as the “AD” group). Those who had a normal biomarker profile or evidence of non-Alzheimer’s pathologic change were classified as being outside the Alzheimer’s continuum (hereafter referred to as the “non-AD” group). Cutoff values for classification of patients within the AD continuum are listed in Supplementary Table S1. As a control group, we included patients with neurological conditions other than dementia or cognitive impairment (mainly suffering from acute or chronic polyneuropathy, multiple sclerosis, or normal-pressure hydrocephalus). For the latter group, we also analyzed the CSF profile of core AD biomarkers. All patients included in the study underwent the collection of unstimulated saliva samples.

Patients with early-stage cognitive impairment or dementia also underwent neuroimaging investigation with magnetic resonance imaging (MRI) or computed tomography (CT) of the brain and neuropsychological evaluation, including the Mini-Mental State Examination (MMSE). Neuroimaging investigations also served to define the “N” criterion of the A-T-N system. Written informed consent for saliva and CSF collection and storage was given by all subjects or by their caregivers. The experiments conformed to the principles of the Declaration of Helsinki and were approved by the local ethics committee with approval protocol number 0114576. 

### 2.2. Saliva Collection

Unstimulated saliva samples were collected from the AD, non-AD, and control groups. All patients were fasting for at least 8 h. All salivary samples were collected immediately after the lumbar puncture. All subjects were asked to refrain from drinking, eating, and smoking for at least 3 h prior to the saliva sampling, which was equivalent to the timing of the lumbar puncture.

Patients were also requested to rinse their mouth with water before providing a 10 mL whole, unstimulated saliva sample in a 15 mL polypropylene Falcon tube. Collected samples were immediately placed on ice and precleared by a low spin at 600 g for 10 min at 4 °C. For determining the Aβ42 levels in saliva samples, fresh saliva was first added to tubes containing 10 µL of thioflavin S (0.5 mg, Sigma, St. Louis, MO, USA) to prevent Aβ42 aggregation. In addition, 2 µL of sodium azide (0.5 mg, Fischer Scientific, Suwanee, GA, USA) was also added to saliva to prevent bacterial growth. Aliquoted 0.5 mL samples were stored at −80 °C. Samples were diluted with 5 µL 4-(2-Aminoethyl)benzenesulfonylfruoride (AEBSF) to a final concentration of 1 mM to prevent proteolysis of Aβ42 peptides. Salivary levels of Aβ42 were quantified by a human Aβ42 ultrasensitive ELISA kit (catalog number KHB3544, only for research use). The standard curve of the Aβ42 ultrasensitive ELISA kit is shown in the Supplementary Materials (Supplementary Figure S6). The human Aβ42 ultrasensitive ELISA quantitates Aβ42 at ultra-low levels in CSF (<1 pg/mL); this kit exclusively recognizes both natural and recombinant Aβ42 US. All samples were assayed in duplicate; this is necessary to account for variation within the assay (intra-assay variation). Preparation of the samples is key in this aspect, and samples should be prepared from stocks where possible to minimize the chance that improper samples will impact final results. Duplicates are also required to provide enough data for statistical validation of the results. The mean values from duplicates were used as a single data point. 

### 2.3. CSF Collection 

Cerebrospinal fluid collection samples were obtained immediately prior to saliva sampling by lumbar puncture using an atraumatic needle, after overnight fasting, and collected in polypropylene tubes. All CSF samples were collected between 9 and 12 a.m. CSF samples were centrifugated at 2500× *g* for 10 min at 4 °C and frozen at −80 °C until analysis. CSF concentrations of Aβ42, t-tau, and tau phosphorylated at 181 were evaluated by a commercial ELISA method with the Innotest Kit for diagnostic use (Fujirebio, catalog number 81574 for p-tau, catalog number 81572 for tau, and catalog number 81576 for Aβ42). 

### 2.4. Statistical Analyses 

All statistics were performed in SPSS version 26.0 (SPSS Inc., Chicago, IL, USA). Continuous variables were described as means and standard deviations, medians, and ranges. Furthermore, categorical variables were defined as count and percentage. Differences in continuous data between groups were studied by the unpaired t-test or its non-parametric variant, the Mann–Whitney test. A chi-square test with Yate’s correction for continuity was used for differences among categorical variables. The correlation between continuous variables was analyzed through the Pearson correlation coefficient or Spearman’s rank correlation coefficient (in the case of a non-normal distribution). To assess the association of CSF Aβ42, t-tau, and p-tau with Aβ42 in saliva, simple linear regressions were performed. The level for statistical significance was set at *p* < 0.05.

## 3. Results

### 3.1. Overall Population

No difference in education, comorbidities, or smoking habits was found among groups. Table 1 shows the demographic and clinical characteristics of the enrolled subjects. 

### 3.2. Analysis of Salivary Aβ42 Concentrations

**Figure 2:**
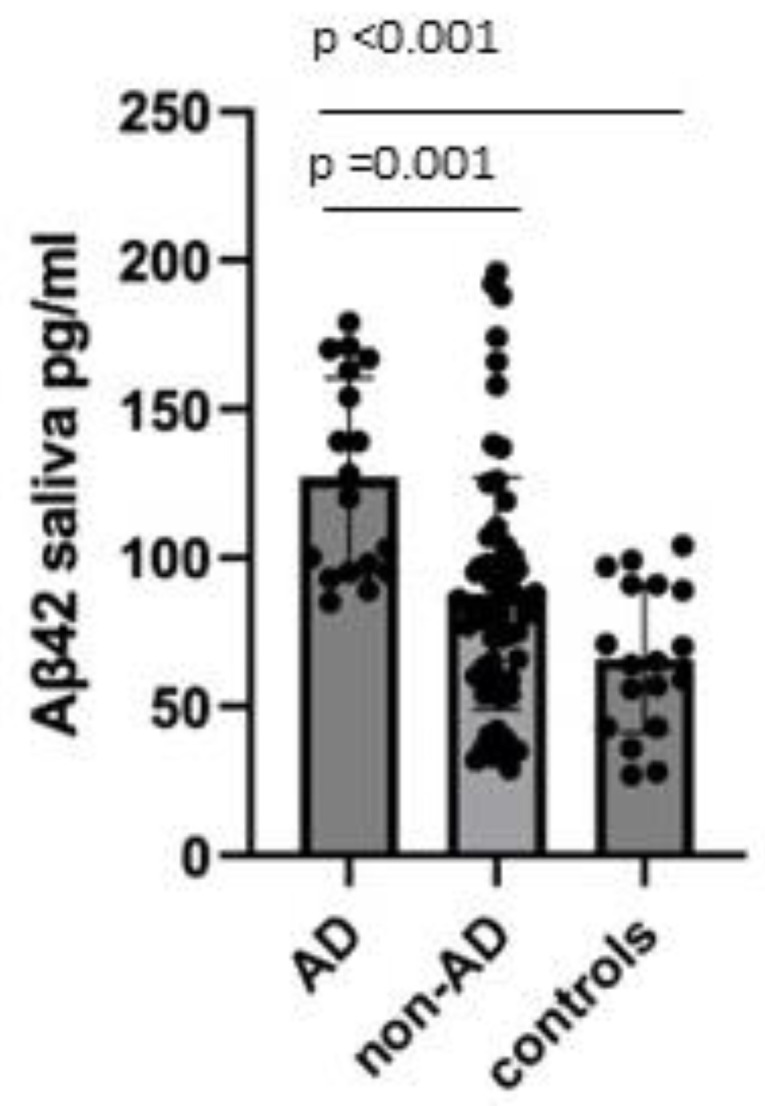
Comparison of salivary Aβ42 concentrations between AD, non-AD, and controls. Data were shown as mean ± SD.

Aβ42 salivary concentrations were found to be significantly increased in AD patients compared to controls (127.11 ± 33.44 pg/mL and 66.11 ± 24.82 pg/mL, respectively; *p* < 0.001), also after adjusting for age (*p* = 0.01) and compared to non-AD (127.11 ± 33.44 pg/mL and 88.03 ± 39.04 pg/mL, respectively; *p* = 0.001) (Figure 2). Aβ42 salivary concentrations were not different between the non-AD group when compared to the controls (88.03 ± 39.04 pg/mL and 66.11 ± 24.82 pg/mL, respectively; *p* = 0.074).

Aβ42 salivary concentrations were not different from males with respect to females (93.61 ± 42.18 pg/mL and 89.42 ± 39.32 pg/mL, respectively; *p* = 0.63) in the three groups (Supplementary Figure S1).

### 3.3. Validation of Diagnostic Performance by the ROC Curve

**Figure 3:**
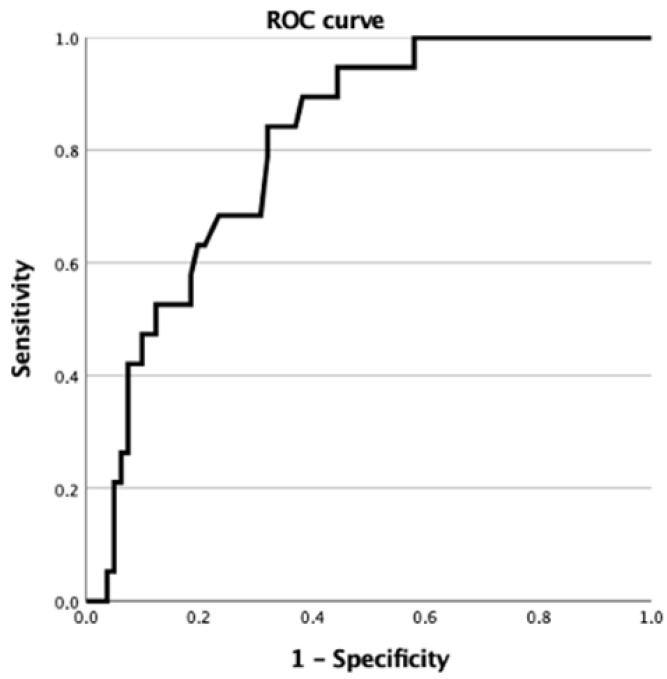
ROC curve of salivary Aβ42 concentrations for the diagnosis of AD.

To calculate the specificity, sensitivity, and accuracy of the saliva compositions for the diagnosis of AD, we identified the ROC curve for the salivary Aβ42 in distinguishing AD from non-AD plus controls. The calculated area under the curve (AUC) of the ROC curve is 0.806. Figure 3 shows the ROC curve of salivary Aβ42 components in the diagnosis of AD. With a cut-off value of 92.5 pg/mL, sensitivity and specificity are 0.84 and 0.68, respectively.

### 3.4. Correlation between Salivary and Cerebrospinal Fluid Concentrations of Aβ42

We hypothesized that the levels of Aβ42 would correlate between salivary and CSF concentrations of Aβ42. CSF Aβ42 concentrations among the three groups were shown in Supplementary Figure S4. 

**Figure 4:**
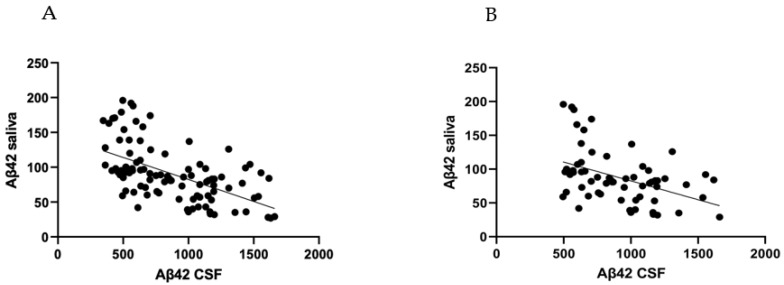

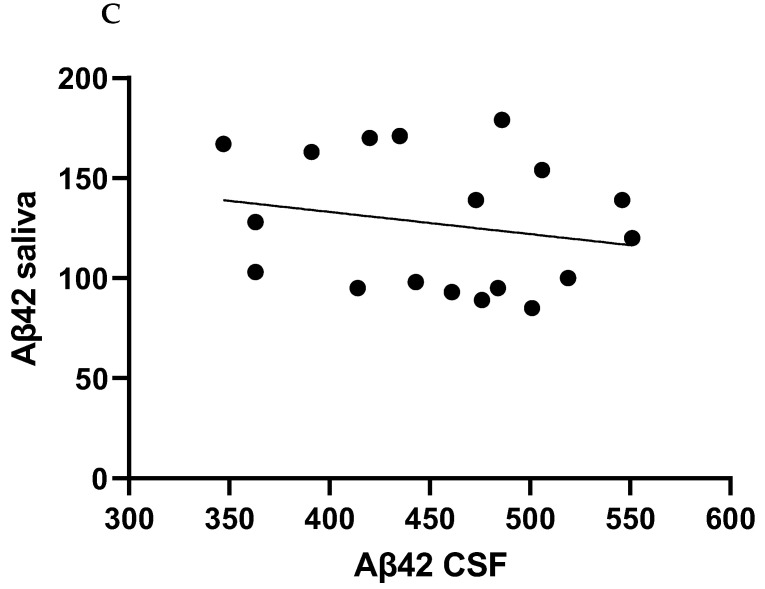
Correlation between salivary Aβ42 concentrations and CSF Aβ42 concentrations (**A**) in the overall population (r = −0.562, *p* < 0.001); (**B**) in the non-AD group (r = −0.443, *p* < 0.001); and (**C**) in the AD group (r = −0.205, *p* = 0.415).

Salivary Aβ42 concentrations correlated negatively with CSF in the overall population (r = −0.562, *p* < 0.001), also adjusting for age participants (r = −0.534, *p* < 0.001), and in the non-AD subgroup (r = −0.443, *p* < 0.001), also adjusting for age participants (r = −0.438, *p* < 0.001) (Figure 4A–C). Importantly, in the AD group, we did not find an inverse correlation between salivary and CSF levels of Aβ42 (r = −0.205, *p* = 0.415), also after adjusting for age (r = −0.198, *p* = 0.447) (Figure 4). We reported correlations between Aβ42 concentrations with p-tau/t-tau in each group and in the overall group, which are shown in Figure S5 of the Supplementary Material.

### 3.5. Salivary Aβ42 and Clinical Characteristics

No clear correlation between salivary Aβ42 concentrations and MMSE scores in the AD group was found (r = −0.708, *p* = 0.075, Figure S2 in the Supplementary Material). A correlation between age and salivary Aβ42 concentrations in the overall group was found (r = 0.236, *p* = 0.019; data are shown in the Supplementary Material, Figure S3). Finally, no relationship between salivary Aβ42 concentrations and the severity of the disease was found.

## 4. Discussion

To the best of our knowledge, this is the first study that investigated the relationship between salivary and cerebrospinal fluid Aβ42 concentrations in a group of patients with dementia and controls. Our study provided new interesting data. Firstly, we confirmed that salivary Aβ42 concentrations are significantly increased in patients with AD (diagnosed according to A-T-N criteria) compared to controls and non-AD patients. Our data show that salivary Aβ42 concentrations are specifically increased in AD and not in patients with other dementias and could therefore represent a potential disease biomarker. According to the ROC analysis performed for the predictive value of Aβ42 concentrations, the AUC was 0.806. This could suggest that measuring salivary Aβ42 can be used as a biomarker to identify patients with AD. Furthermore, in the overall population examined, we found that salivary Aβ42 levels negatively correlated with CSF Aβ42 concentrations.

Our study confirmed previous published findings showing that salivary Aβ42 was either significantly [13] or markedly [4,14] elevated in patients with AD compared to controls. We also found that AD patients secreted levels of salivary Aβ42 double those of controls, confirming published data in different populations [6,15,16]. Furthermore, a recent systematic review suggested that Aβ42 represents a promising future AD-relevant salivary biomarker [6].

The precise mechanism by which Aβ42 accumulates in saliva is unclear. A potential pathway, yet to be demonstrated, calls into question the abundant salivary gland innervation along which CNS biomarkers might travel [9]. On the other hand, buccal cell degradation of amyloid precursor protein (whose expression is widespread) could possibly explain the presence of Aβ peptides in saliva specimens [10]. Interestingly, Aβ42 appears to be increased in AD patients compared with controls not only in the salivary glands but also in other organs, such as the pancreas, spleen, or kidney [11], leading to the hypothesis of Aβ production as a multi-organ phenomenon in Alzheimer’s disease.

To date, ELISA measurement of Aβ42, t-tau, and p-tau (referred to as an Alzheimer’s signature) in CSF is the most accepted method to diagnose probable AD, with a reported high sensitivity and specificity for the diagnosis of AD, even at an early stage [17]. These biomarkers generally reflect the cerebral tau and Aβ pathology [18]. However, lumbar puncture represents an invasive investigation. Contrariwise, saliva is one of the most accessible body fluids and could represent a new source of biomarkers. The identification of robust and reproducible Aβ42 expression in saliva is of particular importance as it may serve as a potential indicator of AD neuropathology that can be measured with non-invasive techniques. In future studies, it could also be of interest to evaluate whether the controls with salivary levels over a hypothetical cut-off will develop AD pathology with the aim of possible use of salivary Aβ42 as a screening biomarker to identify people at risk for AD.

It has been hypothesized that changes in the cerebrospinal fluid may perhaps be reflected in the saliva [19]. In the overall population, we found an inverse correlation between salivary and CSF levels of Aβ42, confirming that saliva may indirectly reflect the changes in CSF. However, in the subgroup analysis, we found an inverse correlation between salivary and CSF levels only in patients with non-AD dementia or in controls, but not in AD patients. These findings could be explained by the limited sample of AD patients involved in the study or could be caused by an impaired clearance of Aβ42 in the salivary glands. It could be hypothesized that the salivary glands in AD patients may have an increased overall Aβ42 production and a clearance that reaches a maximum, not reflecting a linear correlation with the CSF Aβ42 levels. The finding that unstimulated salivary flow from the submandibular glands is impaired in AD patients has already been published [20]. Finally, some authors speculated that the salivary Aβ42 concentration could relatively increase with the severity of AD [12]. In our samples, we did not clearly find a relation between the salivary Aβ42 levels and the stage of AD progression found by Kim et al. [8].

In view of the limited samples, the results obtained and the consequent conclusions should be interpreted with caution, and further studies are needed to confirm our data. However, this is the first study to include AD patients diagnosed with A-T-N criteria. Secondly, the detection of other biomarkers in saliva other than Aβ42, such as p-tau and t-tau, may represent a potential approach for the early detection of AD. Moreover, in previous studies, no difference in salivary t-tau and p-tau concentrations was found between AD patients and non-AD controls [11,21], suggesting that these two proteins are not good candidate salivary biomarkers. Thirdly, we did not find any correlation between salivary Aβ42 levels and the severity of AD. Further prospective studies could provide new evidence regarding whether salivary Aβ42 concentrations may vary during the course of Alzheimer’s disease. Finally, in our study, we included, as controls, patients with neurological conditions other than dementia, and this could influence the findings.

## 5. Conclusions

In this study, we analyzed Aβ42 concentrations in patients with Alzheimer’s disease, patients with dementia different from AD, and a control group. We showed a statistically significant increase in salivary Aβ42 levels in AD patients compared with the other two groups, confirming previous research findings. For the first time, salivary Aβ42 values were compared with levels of the same biomarker at the CSF level, showing a statistically significant inverse correlation in non-AD dementia patients and the control group but not in the AD patient group. It is still premature to define the role of saliva as a potential source of fluid biomarkers in the Alzheimer’s disease landscape. Further studies are needed to define the role of the salivary Aβ42 concentrations in the pathophysiology of Alzheimer’s disease and to better investigate their correlations with the clinical characteristics of the disease.

## Figures and Tables

**Table 1:** Demographic, clinical, and laboratory parameter data of all enrolled subjects.

Demographic and Clinical Characteristics	AD	non-AD	Controls	*p*-Value	*p*-Value after the Bonferroni Correction
Gender M/F (%)	8/10 (44/56)	30/34 (44/56)	7/11 (39/61)	0.843	-
Age (years ±SD)	72.13 ± 5.45	69.34 ± 7.48	65.67 ± 12.02	0.08	-
Disease duration (years ±SD)	2.83 ± 2.28	2.65 ± 2.88	-	0.79	-
MMSE (±SD)	22.47 ± 3.78	23.66 ± 3.95	-	0.34	-
CSF and saliva biomarkers	AD	non-AD	Controls	*p*-Value	*p*-Value after the Bonferroni correction
t-tau [pg/mL] (±SD)	299.50 ± 317.12	157.48 ± 144.94	86.83 ± 65.75	0.002 **	AD vs. non-AD 0.011 * AD vs. Controls 0.002 **
p-tau [pg/mL] (±SD)	60.11 ± 43.53	40.8 ± 40.86	26.28 ± 12.80	0.025 *	AD vs. non-AD 0.18 AD vs. Controls 0.027 *
Aβ42 CSF [pg/mL] (±SD)	454.39 ± 51.86	903.28 ± 311.60	1127.61 ± 358.85	0.000 ***	AD vs. non-AD <0.001 *** AD vs. Controls <0.001 ***
Aβ42 saliva [pg/mL] (±SD)	127.11 ± 33.44	88.03 ± 39.04	66.11 ± 24.82	0.000 ***	AD vs. non-AD <0.001 *** AD vs. Controls <0.001 ***

The following codes define different significance levels: “***” for *p* < 0.001; “**” for *p* < 0.01; and “*” for *p* < 0.05.

## Data Availability

Not applicable.

## Supplementary Materials

The following supporting information can be downloaded at: https://www.mdpi.com/article/10.3390/brainsci12121729/s1, Figure S1: Comparison of salivary Aβ42 concentrations between males and female in AD, non-AD and controls; Figure S2: Correlation between salivary Aβ42 concentrations and MMSE scores in AD group (r = −0.708, *p =* 0.075); Figure S3: Correlation between age and salivary Aβ42 concentrations in overall group (r = 0.236, *p =* 0.019); Figure S4: Comparison of CSF Aβ42 concentrations between AD, non-AD and controls groups; Figure S5: Correlation between salivary Aβ42 and t-tau and p-tau in A) AD group (t-tau r = −0.172, *p =* 0.496), (p-tau r = 0.000, *p =* 0.999), B) non-AD group (t-tau r = 0.408, *p =* 0.001), (p-tau r = 0.267, *p =* 0.033), C) controls (t-tau r = 0.140, *p =* 0.580), D) overall population (t-tau r = 0.321, *p =* 0.001), (p-tau r = 0.297, *p =* 0.001); Figure S6: Example of a calibration curve of Aβ42 ultrasensitive ELISA kit.
