# Supplementary material for: Aβ42 as a Biomarker of Alzheimer’s Disease: Is Saliva a Viable Alternative to Cerebrospinal Fluid?
Source: Brain Sci. 2022 Dec 17;12(12):1729. doi: 10.3390/brainsci12121729 (PMC9775629; doi:10.3390/brainsci12121729)
Supplement: Supplementary file 1 [file brainsci-12-01729-s001.zip › brainsci-2057104-supplementary.pdf]

brain sciences

MDPI

Supplementary Material

Supplementary Figures

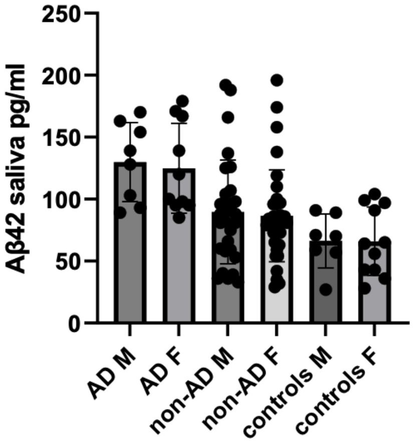

Supplementary Figure S1. Comparison of salivary Aβ42 concentrations between males and female in AD, non-AD and controls.

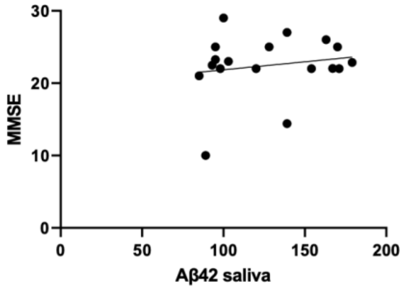

Supplementary Figure S2. Correlation between salivary Aβ42 concentrations and MMSE scores in AD group (r= -0.708, p= 0.075).

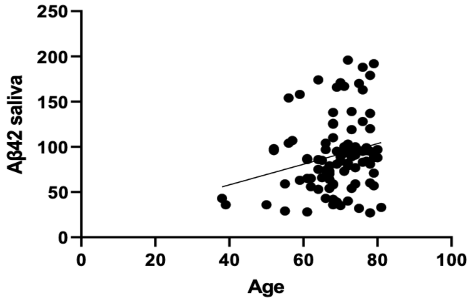

Supplementary Figure S3. Correlation between age and salivary Aβ42 concentrations in overall group (r= 0.236, p= 0.019).

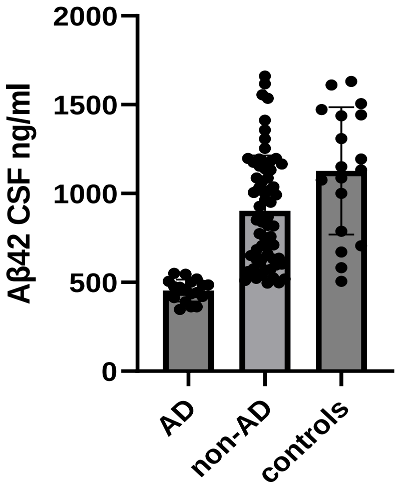

Supplementary Figure S4. Comparison of CSF Aβ42 concentrations between AD, non-AD and controls groups.

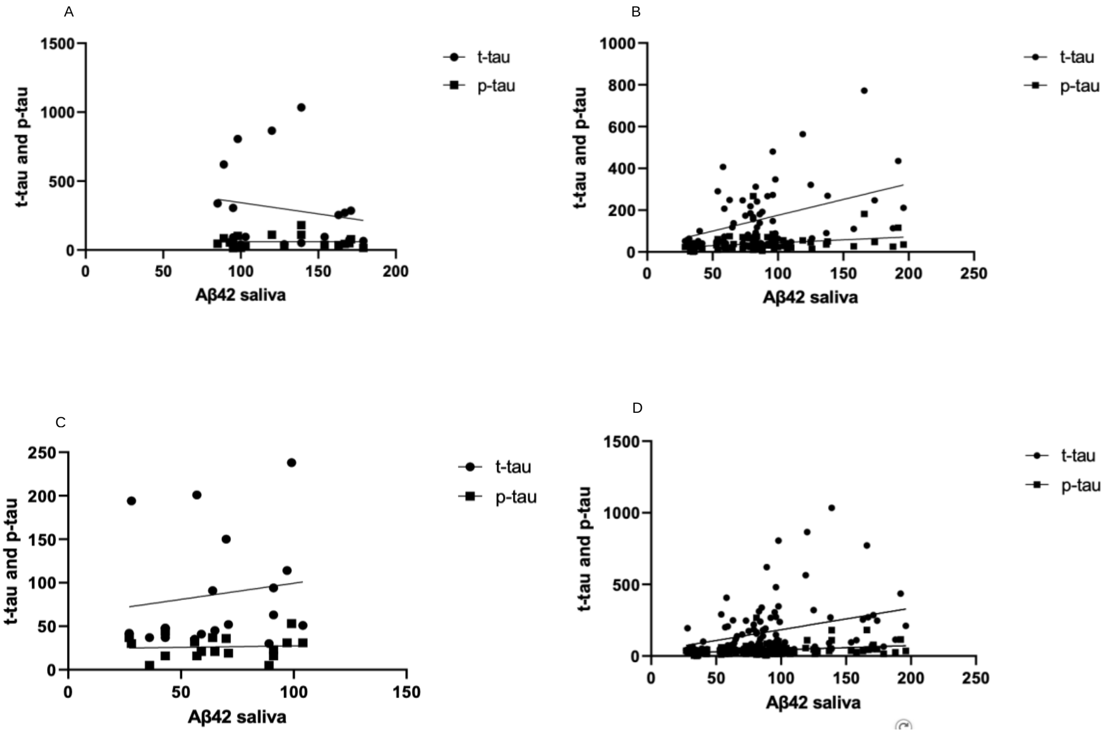

Supplementary Figure S5. Correlation between salivary Aβ42 and t-tau and p-tau in A) AD group (t-tau r= -0.172 , p= 0.496), (p-tau r= 0.000, p= 0.999), B) non-AD group (t-tau r= 0.408, p= 0.001), (p-tau r= 0.267 , p= 0.033), C) controls (t-tau r= 0.140 , p= 0.580), D) overall population (t-tau r= 0.321 , p= 0.001), (p-tau r= 0.297 , p= 0.001).

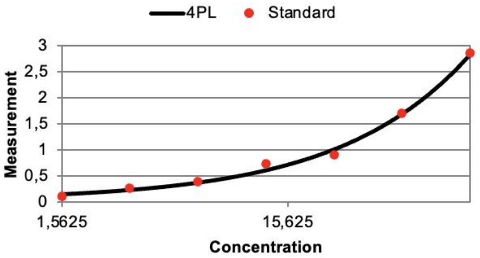

a	0,03081
b	0,76
c	190000
d	876,5
MSE	0,003978
R²	0,9952
SS	0,02785
SYX	0,09635

Calibrator	Conc.	Well	Raw (Corrected)	Backfit
Standard1	100	A1	2,85	100,4
Standard2	50	B1	1,7	50,17
Standard3	25	C1	0,911	21,6
Standard4	12,5	D1	0,729	15,92
Standard5	6,25	E1	0,383	6,468
Standard6	3,125	F1	0,251	3,486
Standard7	1,263	G1	0,016	0,7026

Supplementary Figure S6. Example of a calibration curve of Aβ42 ultrasensitive ELISA kit.
